# Guidelines for Familial Adenomatous Polyposis (FAP): challenges in defining clinical management for a rare disease

**DOI:** 10.1007/s10689-025-00462-y

**Published:** 2025-04-07

**Authors:** Benjamin Zare, Kevin J. Monahan

**Affiliations:** 1 St Mark’s Centre for Familial Intestinal Cancer, St Mark’s Hospital, London, UK; 2 Department of Surgery and Cancer, Imperial College London, London, UK; 3 The St Mark’s Centre for Familial Intestinal Cancer, St Mark’s: The National Bowel Hospital, Central Middlesex Hospital Site, Acton Lane, Park Royal, London, NW10 7NS UK

**Keywords:** Polyposis, Guideline, Colorectal Cancer, Endoscopy

## Abstract

Recent updated management guidelines for Familial Adenomatous Polyposis (FAP) have been published by professional bodies internationally. These recommendations reflect the diverse needs and capabilities of varying health systems worldwide, including thresholds for intervention and population health priorities. Whilst guidelines are closely aligned in many regards, there are areas of disparity. However, alongside discrepancies in guideline recommendations, common challenges also face professional bodies across the globe. Generation of a robust evidence-base in the environment of limited data is difficult in rare diseases such as FAP, underscored by the fact that expert consensus opinion underpins virtually all guidelines. The presence of a wide phenotypic spectrum in FAP and the other hereditary gastrointestinal polyposis syndromes, whilst now well recognised, further complicates the creation of universal recommendations. In this review we draw comparison between the various international guidelines for the management of FAP, using examples to focus on thematic areas of agreement and divergence. However, beyond this, we also wish to highlight the persisting evidence gaps in clinical management, and any areas of ongoing debate among clinicians, where we are yet to establish the optimal approach.

## Introduction

Familial Adenomatous Polyposis (FAP) is an autosomal dominant Mendelian cancer syndrome that is typically characterised by the development of multiple (classically hundreds to thousands) adenomas in the colon and rectum. The phenotype usually manifests from the second decade of life, with early-age colorectal cancer (CRC) in almost all individuals with the condition if it is not identified/treated at an early stage [1]. In modern practice, following the widespread adoption of prophylactic colonic surgery, it is uncommon for patients with FAP to present with CRC, however, there has been wider recognition of phenotypic heterogeneity and evolution in clinical challenges in an aging population reflected in recent clinical guidelines [2].

Within the last decade, updated management guidelines have been published by professional bodies internationally. These guidelines still rely largely on limited data by which clinical management recommendations may be formulated, and are underpinned predominantly by expert opinion. This challenge has been acknowledged in rare diseases through establishment of a database and sharing platform for selection, quality evaluation and dissemination of clinical practice guidelines in rare diseases by organisations such as Orphanet; and, dedicated frameworks for guideline development in rare conditions have been applied to practice guidelines outside of hereditary gastrointestinal polyposis syndromes (e.g., haemophilia A, sickle cell anaemia) with some success [3–6].

Additionally, guideline recommendations should reflect the needs of different health systems, including thresholds for intervention, contribute to variation in the approach to clinical care. Conversely guideline clinical recommendations remain relatively closely aligned in most respects, underpinned by international expert consensus.

This review compares and contrasts these guidelines, identifying commonalities and areas of consensus, as well as a thematic review of areas of controversy and differing approaches where further work is needed to determine the optimal strategy.

This comprises a comparison of the following guidelines (included in summary tables):

1. National Comprehensive Cancer Network (NCCN) Clinical Practice Guidelines in Oncology (NCCN Guidelines®) Genetic/Familial High-Risk Assessment: Colorectal, Endometrial, and Gastric, Version 3.2024, published in 2024 [7].
2. Genetic Testing and Management of Hereditary Gastrointestinal Cancer Syndromes by the American College of Gastroenterology (ACG), published in 2015 [8] (Although largely superseded by NCCN).
3. Endoscopic management of polyposis syndromes: European Society of Gastrointestinal Endoscopy (ESGE) Guidelines, published in 2019 [9].
4. Updated European guidelines for clinical management of familial adenomatous polyposis (FAP), *MUTYH*-associated polyposis (MAP), gastric adenocarcinoma proximal polyposis of the stomach (GAPPS) and other rare adenomatous polyposis syndrome by European Herditary Tumor Group and European Society of Coloproctology (ESCP), published in 2024 [10].
5. Guidelines for the management of hereditary colorectal cancer from the British Society of Gastroenterology (BSG)/Association of Coloproctology of Great Britain and Ireland (ACPGBI)/United Kingdom Cancer Genetics Group (UKCGG), published in 2019 [11].
6. Japanese Society for Cancer of the Colon and Rectum (JSCCR) guidelines 2020 for the Clinical Practice of Hereditary Colorectal Cancer, published in 2020 [12].
7. Management of familial adenomatous polyposis in children and adolescents: Position paper from the ESPGHAN polyposis working group [13].

## Diagnosis: defining uncertainty in FAP

Historically, hereditary GI polyposis syndromes were diagnosed according to the fulfilment of clinical criteria alone. In FAP, this was historically defined as presence of ≥ 100 colon/rectal adenomas. Conversely, although clinical phenotype may inform variant interpretation, a genetic diagnosis relies on confirmation of an underlying pathogenic variant (PV) of a gene associated with the condition – *APC* in the case of FAP. In the past, fulfilment of clinical criteria represented the trigger for genetic testing.

In recent years, studies have confirmed the feasibility and clinical usefulness of multi-gene panel testing (MGPT) and as a consequence the thresholds for genetic testing have been lowered and a number of clear genotype–phenotype correlations have emerged [14–16]. Genetic diagnosis has several distinct advantages including exclusion of other polyposis syndromes, as well as access to interventions such as preimplantation genetic testing (PGT) and research studies.

Crucially a genetic diagnosis facilitates cascade testing in relatives, which results in many avoiding unnecessary intervention when otherwise offered surveillance as a relative of a proband with a clinical diagnosis of FAP. In UK guidelines, for example, a first degree relative of a person with a clinical diagnosis of FAP (i.e., an ‘at-risk’ relative) would require 5 yearly colonoscopy from age 12–14 years.; and, in the US, this would be as frequently as biennially from the late teens.

With ascertainment through MGPT, milder forms of the spectrum are being recognised in patients who would not have been offered genetic testing until recent years. Consequently, we now appreciate the phenotypic spectrum of FAP is broader than the classical presentation, and understand that we were likely missing cases with our historic approach to genetic testing. Although clinical interventions are primarily determined by phenotype in FAP, specific *APC* gene variants may inform risk prediction for cancer or desmoid disease. Conversely, recognition of an FAP phenotype with a very low polyp burden itself (and integration of this in different ways into guidelines) generates clinical challenges, such as the presence of < 10 adenomas not being defined as a clinical diagnosis of FAP.

The ACG and NCCN permit a low adenoma burden of > 10 cumulative colorectal adenomas. Other guidelines recommend higher, or more complex thresholds, given the trade-off between diagnostic yield, resource and effectiveness [17].

The BSG/ACPGBI guidelines recommend an individualised approach to germline testing in the case of multiple colorectal adenomas (MCRA), which they define as presence of 10 or more metachronous colon/rectal adenomas, which reflect a burgeoning but limited evidence base [18, 19]. Where the formal clinical criteria of FAP is not fulfilled, they advise consideration of genetic testing in the instance of a lifetime total of ≥ 10 colonic adenomas under the age of 60, or a lifetime total of ≥ 20 adenomas (or ≥ 10 adenomas plus a family history of either colorectal cancer or polyposis). The JSCCR takes the view that “typical FAP” can be diagnosed from clinical symptoms lone and that genetic testing is not routinely necessary, but that genetic testing (including *APC*) instead can be carried out in the circumstance that either attenuated FAP (AFAP) has to be differentiated from *MUTYH*-associated polyposis (MAP) and polymerase proofreading-associated polyposis (PPAP), or that a patient prefers to undergo genetic testing (to either inform their own treatment or for the purpose of diagnosis in relatives). (see Table 1).

**Table 1:** Summary of FAP clinical diagnostic criteria and recommended genetic testing of ACG, NCCN, ESGE, BSG/ACPGI, and JSCCR

USA (ACG 2015 and NCCN 2024)		Europe (ESGE 2019) and UK (BSG/ACPGBI 2019)		Japan (JSCCR 2020)	
Clinical Diagnostic Criteria	Genes tested	Clinical Diagnostic Criteria	Genes tested	Clinical Criteria	Genes tested
ACG Personal history of > 10 cumulative colorectal adenomas OR Family history of one of the adenomatous polyposis syndromes OR History of adenomas and FAP-type extracolonic manifestations (duodenal/ampullary adenomas, desmoid tumors (abdominal > peripheral), papillary thyroid cancer, congenital hypertrophy of the retinal pigment epithelium) NCCN – > 100 adenomas in colon/rectum (Classic) 10—< 100 adenomas in colon/rectum (Attenuated), although state ‘there is currently no consensus on what constitutes a clinical diagnosis of AFAP’	ACG – *APC, MUTYH* NCCN – In absence of a known pathogenic / likely pathogenic variant, Multi-gene panel testing (including *APC, BMPR1A, EPCAM, MUTYH,* *MLH1, MSH2, MSH6, PMS2, PTEN, SMAD4, STK11, TP53*)	- ≥ 100 adenomas in colon/rectum (Classic) - < 100 adenomas in colon/rectum (Attenuated) (ESGE – ‘… at age 25’)	Multi-gene panel testing (including *APC*, *AXIN2*, *BMPR1A*, *MUTYH*, *MLH1*, *MSH2*, *MSH3*, *PMS2*, *POLE*, *MBD4*, *NTHL1*, *PTEN*, *SMAD4*, *STK11*, *SMAD4*, *RNF43*)	- ≥ 100 adenomas in the large Intestine , irrespective of the presence or absence of a family history of FAP OR - < 100 adenomas in the presence of a family history of FAP	NOT routinely advised^*^

^a^Typical FAP can be diagnosed from clinical symptoms; if a patient prefers to undergo genetic testing for their own treatment/diagnosis in their relatives genetic testing of *APC* is considered, OR if attenuated FAP (AFAP) has to be differentiated from *MUTYH*-associated polyposis (MAP) and polymerase proofreading- associated polyposis (PPAP) then genetic testing (including *APC*, *MUTYH*, *POLE*, *POLD1*) can be considered

*ACG* American College of Gastroenterology, *NCCN* National Comprehensive Cancer Network, *ESGE* European Society of Gastrointestinal Endoscopy, *BSG* British Society of Gastroenterology, *ACPGBI* Association of Coloproctology of Great Britain and Ireland, *JSCCR* Japanese Society for Cancer of the Colon and Rectum, *FAP* Familial Adenomatous Polyposis

### Take home messages

- Though FAP was historically diagnosed clinically, recent advances in MGPT have lowered testing thresholds and revealed broader genotype–phenotype correlations
- Genetic diagnosis offers some distinct advantages: exclusion of other polyposis syndromes, informed risk prediction, cascade testing for relatives, and recognition of milder forms of FAP
- Some guidelines now even recommend individualised genetic testing approaches based on factors such as disease phenotype and family history

## Surveillance and treatment: thresholds for intervention

Population health needs, resources and challenges within health systems vary [20]. Within more socio-economically deprived population and healthcare system, well-described issues such as disparity in patient or clinician education, and systemic barriers within the health service organisation itself, can limit accessibility to genetic testing [21]. As the availability of genetic testing grows across different healthcare economies, the health economic evidence base must also evolve, taking into account different preferences and values.

Beyond diagnosis, the frequency of surveillance and choice of endoscopic/surgical treatment are likely to be affected by the model of healthcare system (universal/population approach, focus on equity of access, individual, insurance-based, etc.) operated within a country, and payment/reimbursement structures within them. And, when attempting to instigate prevention strategies, such as surveillance, there are differing priorities in different healthcare systems, which published work has demonstrated ought to be considered in the design of surveillance recommendation [22].

For example, in more developed countries, there is an older (and ageing) population, which bears relevance when the average age of CRC diagnosis in FAP (without surgical intervention) is 39, and duodenal adenocarcinoma (if it arises) is in the fifties or sixties [23, 24]. The ageing FAP population has increasing complex healthcare needs and multimorbidity, which will limit the appropriateness of surgical and endoscopic intervention [25].

### Take home messages

- Pressures inherent to different healthcare models and reimbursement structures may well account for some observed differences in approach to aspects of surveillance and treatment in FAP
- Among population factors, issues such as socio-economic disparities can restrict guideline approach through resource constraint, whilst others such as advancing population age can limit management in other ways e.g., appropriateness of endoscopic intervention

## Challenges in development of guideline recommendations with limited evidence

Development of clinical guidelines is key to supporting clinical and policy decisions in all global diseases. In principle, guidelines should summarise evidence for outcomes of importance to patients, and then use clear criteria to form recommendations, with evidence with high certainty generating strong recommendations, and low certainty conditional or weak recommendations [26, 27]. However, in the case of rare diseases, such as FAP, guidelines are limited by a lack of high-quality evidence [5].

It is therefore essential that recommendations around diagnostic testing are based on evolving interpretation of the pathogenicity of *APC* variants, and the benefit of interventions in those at lower lifetime risk. Predicated on an association of *APC* I1307K with an intermediate increased risk of CRC (though not FAP per se), the most recent guidelines of the USA’s NCCN recommend that all individuals with such a variant undergo lower gastrointestinal (GI) surveillance by way of colonoscopy at age 40 and then five-yearly thereafter [7, 28]. Whereas, based on a systematic review and meta-analysis of the current evidence (which effectively equates entirely to small sample size studies generating ostensibly inconclusive results regarding cancer risk, particularly in non-Ashkenazi populations) InSiGHT concludes that the I1307K *APC* variant (which it classifies as pathogenic, low penetrance) is a risk factor for CRC in individuals of Ashkenazi Jewish origin only, advising that it should solely be tested for (and carriers offered specific clinical surveillance in) this particular population; in other populations/subpopulations it advises that there is currently insufficient evidence to support an increased risk of cancer, and that those found to have the variant should simply be enrolled in national CRC screening for average-risk individuals [29, 30].

### Take home messages

- Development of clinical guidelines is crucial for informed clinical and policy decisions, particularly in rare diseases like FAP, where high-quality evidence is often lacking
- Recommendations for diagnostic testing, such as the *APC* I1307K variant, must be based on evolving interpretations of pathogenicity and population-specific risks, with differing guidelines reflecting the uncertainty of cancer risk across varying populations

## Endoscopic surveillance according to GI phenotype

Given FAP is a disease with a spectrum of phenotypic presentations, and endoscopic intervals may be tailored to phenotype, with intervals extended in the presence of low polyp burden for example. However, evidence for estimates of risk remain imprecise, being based on historical ascertainment, and therefore a didactic approach to management may not be appropriate, and should be associated with clinical specialist expertise. Thus, most guidelines avoid granular criteria for endoscopic intervention, as it is important to acknowledge that guidelines reflect a limited evidence base, but provide useful guidance. Therefore, more recommendations may provide nuance, expert-led decision-making and individualised care, although alternatively recent European guidelines favour a more directive strategy [31]. However, notably, most guidelines do define an acceptable range with respect to surveillance frequency (e.g., repeat colonoscopy every 1–2 or 1–3 years), which does provide some scope for personalised patient care.

There is a consistent pan-guideline approach to age of initiation and intensity of surveillance (with greater frequency prompted by greater polyp burden). For instance, for classic FAP, lower and upper GI surveillance, respectively, are advised from 10 and 20–25 years of age at 1–2 yearly intervals by the NCCN, as compared to 12 and 25 years of age at 1–3 yearly intervals, according to the BSG/ACGBI, ESGE and ESPGHAN (not included in the summary table). Guidelines now also acknowledge the need for post-colectomy surveillance of the distal colorectum or pouch. Although age/intensity of endoscopic surveillance are comparable, it is worth noting that US guidelines consistently advise slightly tighter/earlier surveillance than other professional bodies for classic FAP.

The Americans’ robust approach with respect to surveillance is also noted in two other scenarios. Post-panproctocolectomy they provide the only explicit recommendation to perform regular ileoscopy in patients. And, in individuals with a first-degree relative (FDR) with a clinical FAP diagnosis in whom an *APC* constitutional PV has not been identified, they recommend relatively frequent colonoscopic surveillance every 2 years; the UK and Europe, by comparison, suggest 5-yearly surveillance (until population screening age. Again, speculatively, this may simply be a function of the types of healthcare systems. and reimbursement structures, at play within different nations. ESGE and BSG/ACPGBI guidelines also explicitly recommend upper GI surveillance in the scenario of a FDR with clinical FAP. (see Table 2).

**Table 2:** Summary of classic FAP endoscopic surveillance recommendations of ACG, NCCN, ESGE, BSG/ACPGBI and JSCCR

	USA (ACG 2015 and NCCN 2024)		Europe (ESGE 2019) and UK (BSG/ACPGBI 2019)		Japan (JSCCR 2020)	
	When to commence	Frequency	When to commence	Frequency	When to commence	Frequency
*APC* pathogenic variant carriers, or clinical diagnosis FAP						
Colonoscopy	10–15 years of age	Every 1–2 years, depending on phenotype	12–14 years of age^a^	Every 1–3 years, depending on phenotype^b^	After the age of 10	1–2 years
Gastroscopy and duodenoscopy	20–25 years of age	As per Spigelman classification	25 years of age	As per Spigelman classification	Not mentioned	As per Spigelman classification
Sigmoidoscopy or pouchoscopy	6–12 months post-colectomy	Every 6–12 months	From time of colectomy	Every 1–3 years, depending on phenotype	From time of colectomy	Long-term surveillance is required after IRA, but frequency not mentioned
Ileostomy	6–12 months post-panproctocolectomy	Annually (NCCN – no specific frequency)	Not mentioned*	Not mentioned	Not mentioned	Not mentioned
Individuals with an FDR with a clinical diagnosis of FAP (i.e., at-risk) and in whom a constitutional pathogenic variant has not been identified (or not tested)						
Colonoscopy	Late teens	Every 2 years, depending on phenotype (NCCN – not mentioned)	12–14 years of age	Every 5 years, until population screening age	Not mentioned	Not mentioned
Gastroscopy and duodenoscopy	Not mentioned	Not mentioned	Commence only if clinical diagnosis made of colorectal polyposis phenotype	As per Spigelman classification	Not mentioned	Not mentioned

EHTG-ESCP (2024) –

^a^In symptomatic patients with germline pathogenic variant in the *APC* gene, OR patients with first-degree relatives affected by classical FAP if a genetic test is not available, OR if no PV is detected, colonoscopy should start at any age and as soon as possible

^b^Repeat endoscopy should be performed within 1 year or less if at least one of the following criteria is present:

-*APC* germline pathogenic variant of codon 1309 associated with a severe phenotype

-Presence of ≥ 100 adenomas at colonoscopy

-Presence of large polyps at colonoscopy (≥ 10 mm)

-Symptoms

-Rapid progression, in terms of polyp size

*ACG* American College of Gastroenterology, *NCCN* National Comprehensive Cancer Network, *ESGE* European Society of Gastrointestinal Endoscopy, *BSG* British Society of Gastroenterology, *ACPGBI* Association of Coloproctology of Great Britain and Ireland, *JSCCR* Japanese Society for Cancer of the Colon and Rectum, *EHTG* European Hereditary Tumor Group, *ESCP* European Society of Coloproctology, *FAP* Familial Adenomatous Polyposis, *IRA* Ileorectal Anastomosis

Regarding milder phenotype disease sometimes called ‘attenuated’ FAP or AFAP, the ESGE and BSG/ACPGBI classifies these with those for classic FAP and determine surveillance intervals on the basis of polyp burden, whereas the NCCN advise a slightly older age of commencement of colonoscopic surveillance of 18–20 years of age (as compared to classic FAP) though recommends the same short interval between surveillance endoscopies [32]. (see Table 3).

**Table 3:** Summary of AFAP endoscopic surveillance recommendations of NCCN, ESGE, EHTG-ESCP, BSG/ACPGBI and JSCCR

Attenuated FAP^a^						
	USA (NCCN 2024))		Europe (ESGE) and UK (BSG/ACPGBI 2019) (as per classic FAP)		Japan (JSCCR 2020)	
	When to commence	Frequency	When to commence	Frequency	When to commence	Frequency
Colonoscopy	18–20 years of age	Every 1–2 years depending on phenotype	12–14 years of age^b^	Every 1–3 years, depending on phenotype	18–20 years	1–2 years
Gastroscopy and duodenoscopy	20–25 years of age	Every 6–12 months, depending on phenotype (NCCN – not mentioned)	25 years of age	Every 1–3 years, depending on phenotype	Not mentioned	Not mentioned
Sigmoidoscopy or pouchoscopy	6–12 months post-colectomy	Every 6–12 months (NCCN – not mentioned)	From time of colectomy	Every 1–3 years, depending on phenotype	Not mentioned	Not mentioned

^a^Diagnostic Criteria for AFAP:

-ESGE: < 100 adenomas in colon/rectum at age 25 (with multi-gene panel testing ruling out other non-*APC* pathogenic variants)

-JSCC: 11–100 adenomas in the large intestine, in absence of constitutional pathogenic variants in genes consistent with *MUTYH*-associated polyposis (MAP) or polymerase proofreading-associated polyposis (*POLE*, *POLD1*)

^b^EHTG-ESCP (2024) –

‘Surveillance can start later but no later than 18–20 years if age in asymptomatic patients with a germline pathogenic variant in the *APC* gene for attenuated FAP disease AND an attenuated proband/family phenotype. If a genetic test is not available or if no known pathogenic variants are detected, surveillance should also begin in asymptomatic patients with first-degree relatives affected by attenuated FAP’

*NCCN* National Comprehensive Cancer Network, *ESGE* European Society of Gastrointestinal Endoscopy, *BSG* British Society of Gastroenterology, *ACPGBI* Association of Coloproctology of Great Britain and Ireland, *JSCCR* Japanese Society for Cancer of the Colon and Rectum, *EHTG* European Hereditary Tumor Group, *ESCP* European Society of Coloproctology, *FAP* Familial Adenomatous Polyposis, *IRA* Ileorectal Anastomosis

### Take home messages

- FAP exhibits a spectrum of phenotypic presentations, and endoscopic surveillance intervals are tailored to phenotype; however imprecise risk estimates lend themself to expert-led and individualised management, rather than rigid guidelines
- Surveillance strategies differ slightly across guidelines, with consistent approaches regarding age and intensity of surveillance, however US guidelines do generally tend to recommend tighter surveillance intervals

## Quality and advanced endoscopic imaging in colonoscopic surveillance

High quality endoscopic surveillance delivered by specialist endoscopists is crucial in order to facilitate accurate non-surgical decision-making, recognise lesions, and identify appropriate timing and approaches to surgical resection (Table 4). Uniquely, the BSG/ACPGBI strongly recommend that all surveillance colonoscopies are performed by endoscopists who consistently achieve BSG colonoscopy KPIs (key performance indicators) minimum standards, as well as recommending a repeat colonoscopy ‘performed by an expert’ in the event of a previously failed colonoscopy. They also advise no advantage is conveyed by advanced endoscopic imaging by chromoendoscopy (either virtual or dye-based), with high-definition white light endoscopy the recommended modality [33]. Although, they do highlight how crucial excellent bowel preparation is for a high-quality colonoscopy, the importance of which has been evidenced outside of FAP in general colorectal cancer prevention, by suggesting a repeat procedure within 3 months in the event of either inadequate prep or suboptimal prep with an incomplete procedure [34].

**Table 4:** Summary of recommendations regarding lower and upper gastrointestinal endoscopic intervention in classic FAP for NCCN, ESGE, EHTG-ESCP, BSG/ACPGBI and JSCCR

	USA (NCCN 2024)	Europe (ESGE 2019)	UK (BSG/ACPGBI 2019)	Japan (JSCCR 2020)
Lower GI endoscopic intervention	No specific thresholds mentioned by either body	Remove all polyps > 5 mm (pre- and post-colectomy)	Not mentioned	Not mentioned
Upper GI endoscopic intervention	NCCN – Duodenal disease: -consider referral to an expert center for management of ‘advanced’ disease -EUS for large adenomas prior to resection (endoscopic or surgical) -ERCP at the time of endoscopic papillectomy Gastric disease: - endoscopically resect polyps ≥ 10 mm OR polyps with endoscopic markers of advanced pathology or high-risk features OR all polyps in the antrum - refer to specialized centers in high-risk polyps for consideration of ESD versus surgery if suspicion of malignancy in a lesion OR lesion cannot be removed by standard techniques (snare or EMR) (ACG – no specific thresholds mentioned)	Duodenal disease: - Non-ampullary adenoma^a^: consider endoscopic resection of adenomas ≥ 10 mm - Ampullary adenoma^b^: consider discussing endoscopic treatment in a MDT setting for adenomas ≥ 10 mm OR showing excessive growth OR with suspicion of invasive growth Gastric disease^c^: *-* Gastric adenomas^c^: resect all that are feasible endoscopically - Fundic Gland Polyps: resect any that are large or symptomatic only (after expert evaluation)	Duodenal disease: - Non-ampullary adenoma: consider endoscopic therapy when Spigelman stage III (annual surveillance also advised) or IV (where surgical therapy should be considered as an alternative) - Ampullary adenoma: Not thresholds/criteria for intervention described, however guideline document states ‘appears to be less safe’ than surgical intervention Gastric disease: Not mentioned	Not mentioned

EHTG-ESCP (2024) –

^a^Non-papillary duodenal lesions measuring 5–10 mm in size could undergo either endoscopic resection or surveillance

^b^All papillary adenomas should be candidates for endoscopic resection, but especially if harbouring HGD, villous histology, or if > 10 mm in size

^c^All gastric adenomas > 5 mm should undergo endoscopic resection

*NCCN* National Comprehensive Cancer Network, *ESGE* European Society of Gastrointestinal Endoscopy, *BSG* British Society of Gastroenterology,*, ACPGBI* Association of Coloproctology of Great Britain and Ireland, *JSCCR* Japanese Society for Cancer of the Colon and Rectum, *EHTG* European Hereditary Tumor Group, *ESCP* European Society of Coloproctology, *GI* Gastrointestinal, *EUS* Endoscopic ultrasonography, *ERCP* Endoscopic retrograde cholangiopancreatography*, ESD* Endoscopic submucosal dissection*, EMR* Endoscopic mucosal resection*, MDT* Multidisciplinary Team

### Take home messages

- High-quality endoscopic surveillance is essential for accurate decision-making and appropriate surgical timing in FAP
- BSG/ACPGBI guidelines uniquely emphasise this in their recommendations – white light endoscopy (over advanced imaging) and repeat colonoscopy if bowel preparation is inadequate or procedure is suboptimal

## Indications for colectomy and choice of surgical procedure

Although largely in harmony, there remain key differences in guideline recommendations in the extent of resection for people with FAP (Tables 5 and 6). These reflect variable perspectives about the consequences of surgery to patients including morbidity, quality of life, and cancer risk. The ACG, BSG/ACPGBI and EHTG-ESCP guidelines categorise recommendations for surgery into ‘absolute’ and ‘relative’ indications for colorectal resection. Criteria for immediate surgery include cancer and severe polyposis phenotypes, whereas relative indications (or criteria for planned surgery), which include moderate or accelerating (on surveillance) polyp burden. Similarly, both the ACG and EHTG-ESCP define comparable criteria for choice of surgery – colectomy with ileorectal anastomosis (IRA) versus proctocolectomy with ileal pouch-anal anastomosis (IPAA) also known as restorative proctocolectomy (RPC) – focused on the burden of rectal polyps. However, the EHTG-ESCP criteria for immediate surgery are broader, e.g., the presence of a single polyp with high-grade intra-epithelial neoplasia (HGIEN) or villous histology.

The BSG/ACPGBI guidelines recognise the balance between nuance and specificity, in their approach to selecting the anatomical extent of prophylactic colonic surgery in patients with FAP. They potentially widen the criteria for the less morbid total colectomy with ileorectal anastomosis, and emphasise that flexibility should be employed with regard to polyp number thresholds, given the advent of modern high-definition/chromo-endoscopic techniques [11].

The JSCCR guidelines meanwhile divide recommendations for surgery based on intention – cancer prevention or cancer treatment. Notably, however, with respect to the latter (which naturally represents the majority of surgical cases in FAP), the JSCCR recommends IPAA as the standard surgical procedure of choice.

**Table 5:** Summary of recommendations regarding lower gastrointestinal surgical intervention in classic FAP for ACG, NCCN, EHTG-ESCP, BSG/ACPGBI and JSCCR

USA (ACG 2015)^a^		Europe (EHTG-ESCP 2024)		UK (BSG/ACPGBI 2019)		Japan (JSCCR 2020)	
Absolute indication	Relative indication	Immediate surgery	Planned surgery	Absolute indication	Relative indication	Cancer prevention	Cancer treatment
documented or suspected cancer OR significant symptoms * Colectomy with IRA if:* < *20 rectal AND/OR* < *1000 colonic adenomas* * TPCwith IPAA if:* > *20 rectal adenomas AND/OR* *1000 colonic adenomas*	- presence of multiple adenomas > 6 mm OR - significant increase in adenoma number OR - inability to adequately survey the colon because of multiple diminutive polyps	- certain or suspected cancer - severe symptoms - “severe disease” (> 1000 polyps at colonoscopy) OR - unfavourable histologic polyp features (HGIEN or villous adenoma) *Colectomy with IRA* */near-total colectomy if:* ≤ *5 rectal adenomas AND* < *500 colic adenomas* *TPC with IPAA if:* *around 500 /more colonic adenomas* *OR* ≥ *20 rectal adenomas* *OR* *APC mutation at codon 1250–1450*	- polyp > 10 mm in diameter with favourable histologic features OR - substantial increase in polyp number between examinations OR - “sparse disease “ (defined as 100–1000 polyps at colonoscopy) *Colectomy with IRA* */near-total colectomy if:* < *20 rectal adenomas AND* *all are less* < *5 mm* ± *all* > *5 mm are removable by endoscopy* *TPC with IPAA if:* *distal rectal cancer OR* *cancers requiring radiation* *OR* *incontinence/* *poor sphincter function* *OR* *those who desire to avoid the functional consequences of an IPAA*	- polyp > 10 mm in diameter OR - high grade dysplasia within polyps OR - a significant increase in polyp burden between screening examinations		most patients should undergo colonic surgery in their 20 s and some in their teens; currently, IPAA is considered the standard surgical procedure for prophylactic surgery	If curative resection can be expected, IPAA or IRA with dissection of the regional lymph nodes is an option; if cancer cannot be curatively resected, a surgical procedure such as that for sporadic colorectal cancer should be selected

^a^NCCN (2024)

No division into absolute or relative indications for surgery, and no precise criteria regarding choice of Colectomy with IRA vs. TPC with IPAA, however –

NCCN indications for proctocolectomy with end-ileostomy:

very low advanced rectal cancer OR inability to perform IPAA OR patient with IPAA with unacceptable function OR patient with a contraindication to IPAA OR concern regarding ability to participate in close endoscopic surveillance after surgery OR patient choice

*ACG* American College of Gastroenterology, *NCCN* National Comprehensive Cancer Network, *EHTG* European Hereditary Tumor Group, *ESCP* European Society of Coloproctology, *BSG* British Society of Gastroenterology, *ACPGBI* Association of Coloproctology of Great Britain and Ireland, *JSCCR* Japanese Society for Cancer of the Colon and Rectum, *HGIEN* High-grade intra-epithelial neoplasia, *TPC* Total proctocolectomy, *IRA* Ileorectal anastomosis, *IPAA* Ileal pouch-anal anastomosis

**Table 6:** Summary of recommendations regarding upper gastrointestinal surgical intervention in classic FAP for ACG, ESGE, EHTG-ESCP, BSG/ACPGBI and JSCCR

USA (ACG 2015 and NCCN 2024)	Europe (ESGE 2019)	UK (BSG/ACPGBI 2019)	Japan (JCCR 2020)
ACG – Curative resection: duodenal adenocarcinoma *(Pancreato-duodenectomy recommended)* Spigelman IV duodenal polyposis with involvement of duodenal papilla *(Pancreato-duodenectomy recommended)* Spigelman IV duodenal polyposis without involvement of duodenal papilla (*Pancreas-preserving duodenectomy candidate)* (NCCN – Spigelman IV only) NCCN absolute criteria for gastric surgery: multifocal high-grade dysplasia OR Intramucosal/invasive cancer (ACG – not mentioned)	Curative resection^a^ - histologically proven duodenal (including papillary) or gastric adenocarcinoma, if surgically resectable *(Pancreato-duodenectomy recommended)* - Spigelman III-IV duodenal polyposis without invasive tumour, where endoscopic downstaging is no longer manageable *(Pancreato-duodenectomy recommended)* Prophylactic resection^b^ -Spigelman IV duodenal polyposis -Spigelman II-III duodenal polyposis that is not endoscopically manageable	No explicit indications described but suggests consideration of surgery for Spigelman IV duodenal polyposis (or alternatively endoscopic intervention, if appropriate)	No explicit indications described but suggests that for Spigelman IV duodenal polyposis, one should carry out 6–12 monthly ‘assessment of the indication for surgery’ by a specialist

EHTG-ESCP (2024) recommendations regarding gastric surgery:

^a^Curative gastrectomy criteria: gastric intramucosal carcinoma OR adenocarcinoma

^b^Prophylactic gastrectomy criteria (consideration of): proximal polypoid mounds (fundic gland polyp, pyloric gland adenoma, or tubular adenoma) AND high-grade dysplasia) AND high-grade dysplasia

*ACG* American College of Gastroenterology, *NCCN* National Comprehensive Cancer Network, *ESGE* European Society of Gastrointestinal Endoscopy, *BSG* British Society of Gastroenterology, *ACPGBI* Association of Coloproctology of Great Britain and Ireland, *JSCCR* Japanese Society for Cancer of the Colon and Rectum, *EHTG* European Hereditary Tumor Group, *ESCP* European Society of Coloproctology,

### Take home messages

- Guidelines regarding surgery vary between professional bodies, and differences in defining absolute and relative indications for surgery reflect an attempt at balance by guidelines between morbidity, quality of life, and cancer risk
- In terms of colonic surgery, the ACG, BSG/ACPGBI, and EHTG-ESCP define comparable criteria for respective surgical approaches (though with some variation regarding immediate surgery)
- The JSCCR is an outlier in universally favouring total proctocolectomy IPAA as its preferred prophylactic surgery

## Extracolonic manifestations of FAP

People with FAP are at an elevated risk for development of papillary carcinoma of the thyroid (PTC). Given this, ACG guidelines unambiguously recommend annual ultrasonographic surveillance for PTC in FAP patients. However, there is limited evidence of the effectiveness of this approach, and work from the Cleveland Clinic having suggested that annual ultrasound may be unnecessary in the context of a normal baseline scan (suggesting consideration of extension to two-yearly surveillance until nodules are detected), most other guidelines suggest a more prudent approach [35, 36]. European (paediatric) and Japanese guidelines do not favour routine thyroid surveillance in FAP [12, 13].

### Take home messages

- European and Japanese guidelines do not endorse routine thyroid monitoring for FAP patients, based on limited available evidence
- The ACG, contrastingly, suggests annual thyroid ultrasound

## Conclusions and future directions

As for other rare syndromes, guideline recommendations for FAP are underpinned by expert opinion. Although guideline development may incorporate a systematic review of the literature, and recommendations developed through a Delphi consensus process, the data on which the questions have been formed are predominantly observational, and therefore expertise is required in their formulation. There are small numbers of notable RCTs with limited numbers of patients (e.g., COX-2 inhibition use for attempted reduction in polyp burden, and nirogacestat administration for treatment of desmoid tumours) [37, 38]. As we encounter ever more complex questions for ever smaller subsets of these patient groups (e.g., those with particular genotypes or phenotypes, or aging patient populations) it may be necessary to adapt our approach to evidence generation through the development of research priorities, and greater levels of international collaboration, led by experts in the condition.

Some guidelines suggest centralisation of services and consideration of holistic elements of care.[11] This approach underlies the development and implementation of Rare Disease Collaborative Networks (RDCNs) in England – recognised networks of member providers (Rare Disease Collaborative Centres) with an evidenced research-active interest in a rare disease – with the aim of improving patient outcomes, an example of which that has been implemented with success across the UK is the RDCN for Hereditary Gastrointestinal Polyposis Syndromes [39].

There remain areas of clinical management that are largely unaddressed compared to historical FAP populations. Although extra-colonic manifestations, specifically desmoid disease and duodenal adenocarcinoma, are now the chief disease-related causes of mortality in FAP, and that at least one study has described a marked increase in the incidence of gastric cancer in FAP within the last decade, their management remains poorly addressed in guidelines [40–42]. Clinicians require the evidence, guidelines and infrastructure to manage patients with an evolving and broad spectrum of disease, or an evolving morbidity spectrum which reflects a more clinically effective approach to management of colorectal or duodenal disease and therefore an aging FAP population.

## Data Availability

No datasets were generated or analysed during the current study.
